# Dedifferentiated Leiomyosarcoma of the Auricle with Heterologous Osteosarcoma Component: Case Report and Literature Review

**DOI:** 10.1155/2022/3684461

**Published:** 2022-05-31

**Authors:** Andrea Dekanić, Nives Jonjić, Anita Savić Vuković

**Affiliations:** ^1^Department of Pathology and Cytology, Clinical Hospital Center Rijeka, Rijeka, Croatia; ^2^General Pathology and Pathological Anatomy, University of Rijeka, Faculty of Medicine, Rijeka, Croatia

## Abstract

Leiomyosarcomas are rare malignant tumors of smooth muscles. Head and neck involvement by this disease is very rare, and cutaneous leiomoysarcomas of the ear are even rarer. This is way clinically they are usually mistaken for either squamous or basal cell carcinomas, as was the case in an 85-year-old male patient presented in this report. However, the final diagnosis was even more interesting considering that it was a dedifferentiated leiomyosarcoma of the auricle with a heterologous component of osteosarcoma. The auricular cutaneous malignancies have a much higher rate of recurrence than the corresponding malignancy in other regions of the head and neck, even when resected with negative surgical margins, and dedifferentiated leiomyosarcoma is clinically even more aggressive. Thus, the treatment of choice is a total auriculectomy and great attention should be paid to appropriate margins.

## 1. Introduction

Leiomyosarcoma is a malignant mesenchymal neoplasm of smooth muscle origin. As an aggressive soft tissue sarcoma, it is usually found in adult and elderly patients. The most commonly involved sites are extremities, retroperitoneum, abdomen/pelvis, and trunk, accounting for approximately 11% of all newly diagnosed soft tissue sarcomas [1]. Soft tissue sarcomas in the head and neck, in general, are not frequent, they account for less than 10% of all soft tissue sarcomas, and less than 1% of all neoplasm of this region [2, 3].

Leiomyosarcoma of the head and neck represent only 1% to 4% of those tumors [4], and the ear is the further extremely rarer site in the category of head and neck region. The tumor can be superficial in the cutaneous or subcutaneous layer, or it can present in the deeper tissue. Subcutaneous leiomyosarcoma is thought to develop from smooth muscle present in the walls of blood vessels whereas the cutaneous variant is derived from the erector pili musculature of the skin hair [5, 6].

The term “dedifferentiated leiomyosarcoma” refers to a soft tissue leiomyosarcoma containing areas of nonspecific, poorly differentiated, pleomorphic appearance, or morphologically characterized by an abrupt transition from classic leiomyosarcoma to high-grade sarcoma, which does not express immunohistochemical muscular markers [7]. In rare cases, the nonmyogenic component of the tumor presents heterologous osteogenic differentiation. Here, we describe a case, which is to the best of our knowledge the first report in the literature, of cutaneous auricular dedifferentiated leiomyosarcoma with heterologous, osteosarcomatous component.

## 2. Case Report

**Figure 1:**
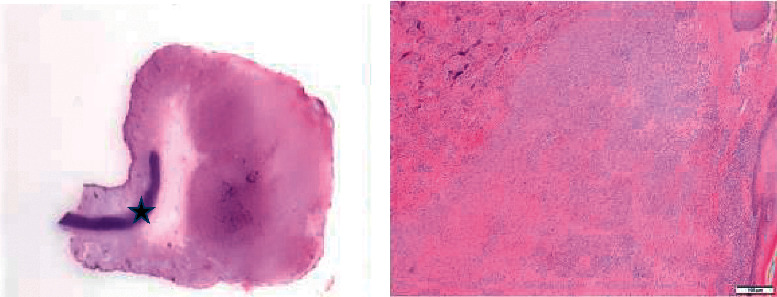
Auricular tumour node with an ulcerated surface that infiltrates the dermis while preserving cartilage (^*∗*^) (a). Transition from differentiated component with smooth muscle morphology to osteosarcoma component (b).

An 85-year-old male patient was admitted to the Clinic for Otorhinolaryngology, due to the removal of a tumor on the right ear. The patient has been in general good condition and has not had any serious illness until then. After an excisional biopsy, the material was examined at the Clinic of Pathology and Cytology. Macroscopic examination showed helix ear tissue with solid tumor mass measuring 1.9 × 1.7 cm, ulcerated surface (Figure 1(a)).

**Figure 2:**
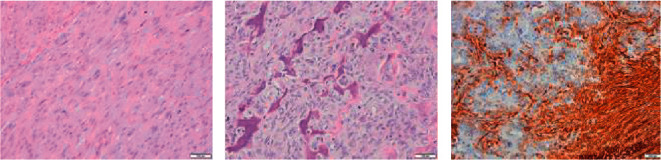
Spindle cell morphology of leiomyosarcoma (a) and dedifferentiated component of tumour characterized by pleomorphic, atypical cell producing osteoid (b) that was immunohistochemically completely negative for smooth muscle actin (SMA, as well as other muscle markers) (c).

Histologically, well-circumscribed dermal cellular nodule showed two different, sharply separated components (Figure 1(b)). One consisted of atypical spindle cells arranged in intersecting fascicles with pleomorphic nuclei, with numerous atypical mitotic figures (more than 20 mitoses/10 HPF). The other component consisted of atypical pleomorphic cells producing osteoid matrix in disorganized trabeculae with focal calcification, and osteoclastic giant cells were frequently scattered throughout the osteoid matrix (Figures 2(a) and 2(b)). Areas of osteosarcoma were located in the center of the tumor. The tumor infiltrated the dermis but not the cartilage. The resection margins were not affected by the tumor.

Additional immunohistochemical analyses with vimentin, *α*-smooth muscle actin, desmin, calponin, pan-cytokeratin (AE1/AE3), and S-100 protein was performed. Areas with leiomyosarcomatous appearance were strongly positive for alpha smooth muscle actin (Figure 2(c)) and vimentin, while focally positive for desmin and calponin, and negative for S-100. The osteosarcomatous component was strongly positive only for vimentin while it was negative for all other markers. Both components were negative for cytokeratin AE1/AE3.

The final diagnosis was leiomyosarcoma grade 2, according to the French Federation of Comprehensive Cancer Centers (FNCLCC) grading system [8] with areas of high-grade osteosarcoma.

After diagnosis, the radical auricular resection was recommended as the final treatment with a regular follow-up that includes examination of the neck lymph nodes.

## 3. Discussion

Leiomyosarcomas of the head and neck are very rare, and usually reported in sinonasal track, oral cavity, tongue, hypopharynx, larynx, trachea, and cervical esophagus [9–12]. Superficial or cutaneous presentation of leiomyosarcoma typically occurs on the face or scalp [13]. Very infrequently these neoplasms can be also found in the ear region. In general, malignant tumors of the ear are very rare, they make up about 7% of all skin tumors, and most of them are carcinomas such as basal cell and squamous cell carcinoma. Still, several reports in the literature describe cases with leiomyosarcoma involved the auricle [14–17], postauricular region [18] and external ear [19].

The clinical presentation in our case was not significantly different from the previously described auricular or cutaneous leiomyosarcoma, as a nodule (firm, skin colored, and with hyperkeratosis) suggestive of a probable squamous cell carcinoma. [20] However, what set this case apart from the usual ones were the histological findings of dedifferentiation within the muscle component. Specifically, the peripheral part of the tumor, confined to the dermis, showed poorly atypical spindle-shaped cells with elongated nuclei and blunted ends, and interlacing fascicles that weave and blend into the collagen, consisted with the morphology of leiomyosarcoma (confirmed immunohistochemically), while central part presented with atypical pleomorphic cells producing osteoid matrix, corresponding to osteosarcoma.

Dedifferentiation is a well-known occurrence in soft tissue tumors such as liposarcoma, chondrosarcoma, paraosteal osteosarcoma, chordoma, and solitary fibrous tumor [21]. Nonetheless; this phenomenon has been rarely reported in leiomyosarcoma. Most often, the dedifferentiation is characterized by an abrupt transition from a low-grade well differentiated neoplasm to the high-grade undifferentiated tumor. Some previous studies have designated leiomyosarcoma with a distinct anaplastic component as “dedifferentiated leiomyosarcoma,” although a subset of these tumors has still retained expression of muscle markers in the poorly differentiated areas [22, 23]. Recently, Nicolas et al. in their large study with 41 cases subclassified leiomyosarcoma with pleomorphic features into two categories: “pleomorphic leiomyosarcoma” with a retained expression of muscle markers, and “dedifferentiated leiomyosarcoma” with a lack of muscle marker expression in the high-grade pleomorphic component [24]. This classification is accepted in the 2020 World Health Organization Classification of Soft Tissue and Bone Tumors [7].

Chen et al. analyzed 18 cases of dedifferentiated leiomyosarcoma, mostly presented in the retroperitoneum, followed by the extremities, the shoulder, visceral organs (uterus and prostate), and the paratesticular region. The dedifferentiated component of leiomyosarcoma simulate the morphology of so-called MFH, rhabdomyosarcoma, heterologous elements showed metaplastic bone and cartilage formation, and in one case osteosarcoma [21]. In our case, the dedifferentiated leiomyosarcoma with osteosarcoma component was presented in the auricle as a primary lesion, while in some cases osteosarcoma dedifferentiation occurred within local recurrence of forearm [25] or groin region soft tissue leiomyosarcoma [26].

Generally, recommended treatment of leiomyosarcoma is primarily surgical and consists of a wide local excision with emphasis on negative margins. For superficial leiomyosarcoma that comprise local excision with a 2 to 5 cm margin, including subcutaneous tissue and fascia [27]. Routine lymph node dissection is not included unless lymph node disease is evident [28]. Treatment failures of leiomyosarcomas in the head and neck region are usually attributable to local recurrence due to the relative difficulty of adequate resection in this region. Thus, reported recurrence rates of cutaneous leiomyosarcomas range from 30% to 50% [29]. According to Pai et al. the auricular cutaneous malignancies, such as melanoma and squamous cell carcinoma, have a much higher rate of recurrence than the corresponding malignancy in other regions of the head and neck, even when resected with negative surgical margins. The authors assume that relatively thin skin overlying the cartilage of the external ear contributes to a wider subclinical extension and horizontal growth along the dermis and perichondrium, often making initial adequate resection difficult. Lesions of the auricle also have a tendency to spread along the neurovascular and embryonic fusion planes of the ear, thereby invading adjacent structures [16]. However, our patient developed dedifferentiated leiomyosarcoma that is clinically even more aggressive [8], with the incidence of metastasis 89% and the mortality rate 50% at the last follow-up [7]. Thus, the treatment of choice is a total auriculectomy and great attention should be paid to appropriate margins. Adequate surgical resection is directly associated with long term survival rate [6].

In conclusion, with the present case, we wanted to point out the possibility of very rare sarcoma in the ear region such as leiomyosarcoma that are generally clinically diagnosed late or misdiagnosed. In addition, this case is particularly interesting since leiomyosarcoma showed dedifferentiation with a heterologous component such as osteosarcoma. This incidence, although very rare, is important to recognize because of its clinical significance in terms of poorer prognosis, which implies an adequate approach to therapy.

## Data Availability

The data used to support the findings of this case report are available from the corresponding author upon request.
